# Experimental Determination of Optimal Conditions for Reactive Coupling of Biodiesel Production With *in situ* Glycerol Carbonate Formation in a Triglyceride Transesterification Process

**DOI:** 10.3389/fchem.2018.00625

**Published:** 2018-12-13

**Authors:** Luma Sh. Al-Saadi, Valentine C. Eze, Adam P. Harvey

**Affiliations:** School of Engineering, Newcastle University, Newcastle upon Tyne, United Kingdom

**Keywords:** reactive coupling, FAME, glycerol carbonate, biodiesel, design of experiment

## Abstract

This study investigated a reactive coupling to determine the optimal conditions for transesterification of rapeseed oil (RSO) to fatty acid methyl ester (FAME) and glycerol carbonate (GLC) in a one-step process, and at operating conditions which are compatible with current biodiesel industry. The reactive coupling process was studied by transesterification of RSO with various molar ratios of both methanol and dimethyl carbonate (DMC), using triazabicyclodecene (TBD) guanidine catalyst and reaction temperatures of 50–80°C. The optimal reaction conditions obtained, using a Design of Experiments approach, were a 2:1 methanol-to-RSO molar ratio and 3:1 DMC-to-RSO molar ratio at 60°C. The FAME and GLC conversions at the optimal conditions were 98.0 ± 1.5 and 90.1 ± 2.2%, respectively, after 1 h reaction time using the TBD guanidine catalyst. Increase in the DMC-to-RSO molar ratio from 3:1 to 6:1 slightly improved the GLC conversion to 94.1 ± 2.8% after 2 h, but this did not enhance the FAME conversion. Methanol substantially improved both FAME and GLC conversions at 1:1–2:1 methanol-to-RSO molar ratios and enhanced the GLC separation from the reaction mixture. It was observed that higher methanol molar ratios (>3:1) enhanced only FAME yields and resulted in lower GLC conversions due to reaction equilibrium limitations. At a 6:1 methanol-to-RSO molar ratio, 98.4% FAME and 73.3% GLC yields were obtained at 3:1 DMC-to-RSO molar ratio and 60°C. This study demonstrates that formation of low value crude glycerol can be reduced by over 90% compared to conventional biodiesel production, with significant conversion to GLC, a far more valuable product.

## Introduction

Fatty acid alkyl esters are usually produced by a transesterification of triglyceride-containing feedstocks (vegetable oils, animal fat etc.) with short chain alcohols. The most commonly used alcohol is methanol due to its low price and availability (Zabeti et al., 2009), and such triglyceride transesterification process produces fatty acid methyl esters (FAMEs) as the main product and glycerol as a by-product. Mixtures of fatty acid alkyl esters (≥96.5% according EN14214) produced during transesterification are used as biodiesel, a renewable alternative to petro-diesel, and this accounts for about 82% of the biofuels production in the EU (Demirbas and Balat, 2006). Conventional biodiesel production uses homogeneous base-catalyzed transesterification process in the presence of alkali metal hydroxides and methoxides (NaOH, KOH, NaOCH_3_, KOCH_3_), especially sodium methoxide which accounts for than 60% of the commercial biodiesel plants (Huber et al., 2006). Base catalysts are the most commonly used methods of catalysis in processing of vegetable oil feedstock containing low levels of free fatty acids (FFAs), due to the faster reaction rates of the base-catalyzed transesterification, typically about 4,000 times faster than acid catalysts at moderate temperatures (Cervero et al., 2008). However, for biodiesel productions from low-grade vegetable oil feedstock containing high levels of FFAs (≥0.5 wt%) and water content above 0.3 wt%, acid catalysts are required to avoid soap formations. Such low-grade feedstock would require a one-stage acid-catalyzed transesterification or a two-stage process involving an acid-catalyzed initial FFA pre-treatment step followed by a base-catalyzed triglyceride transesterification (Canakci and Van Gerpen, 2003; Moser, 2009). Some studies have also shown that organic bases such as guanidine, especially the triazabicyclodecene (TBD) guanidine, are active for catalysis of triglyceride transesterification (Schuchardt et al., 1995; Bromberg et al., 2010). A major advantage of the TBD guanidine over alkali metal hydroxides and methoxides is that it does not cause triglyceride and FAME saponification side reactions (Schuchardt et al., 1995), whereas such side reactions have been reported for homogeneous alkali metal catalysts (Phan et al., 2012; Eze et al., 2014, 2018). The TBD guanidine can also be grafted onto supports such as silica and used as a stable heterogeneous catalyst (Derrien et al., 1998; Sercheli and Vargas, 1999; Meloni et al., 2011; Nguyen et al., 2013).

In conventional triglyceride transesterification, crude glycerol constitutes about 10–20% (v/v) of the product stream (Ayoub and Abdullah, 2012; Quispe et al., 2013). This crude glycerol by-product needs to be either purified for further use or discarded as a waste which leads to environmental problems. The co-production of crude glycerol in the conventional biodiesel processes has little economic advantage for biodiesel plants, as the huge rise in global glycerol production has caused its oversupply, significantly reducing the glycerol price (Rodrigues et al., 2012). It has been predicted that the worldwide glycerol surplus will rise to over 6 million tons in 2025 (Ciriminna et al., 2014). Therefore, it is important to find a way to upgrade the glycerol into valuable chemicals such as 1,3-propanediol (Mu et al., 2006), citric acid (Papanikolaou et al., 2002), polyhydroxyalkanoates (PHA) (Ashby et al., 2004), solketal (Mota et al., 2010; Eze and Harvey, 2018), and glycerol carbonate (GLC) (Esteban et al., 2015; Ishak et al., 2016). It has been reported that one of the most promising processes for valorisation of glycerol is through conversion to GLC, a valuable chemical in industrial productions of polymers and a non-toxic electrolyte for batteries (Ishak et al., 2016). GLC is also classed as a “green” solvent and significantly more valuable than glycerol. It is usually produced by reacting glycerol with DMC using a base catalyst (Teng et al., 2016). Bulk productions of GLC is envisaged in from a glycerol output of substantially large biodiesel plants, hence, *in situ* conversions of the glycerol by-product to GLC inside the transesterification reactor would be of potential advantage. This would minimize process costs for additional steps required for crude glycerol purification and subsequent valorisation.

**Figure 1:**
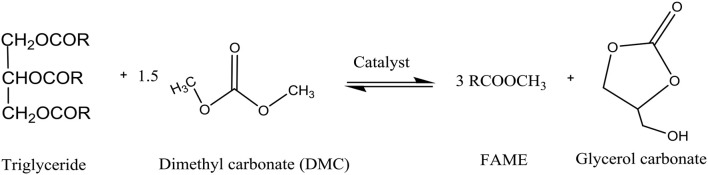
Reaction of triglyceride and DMC.

Consequently, there has been significant recent research interest in replacement of methanol with dimethyl carbonate (DMC) in biodiesel production reactions to minimize crude glycerol production (Zhang et al., 2010; Seong et al., 2011; Rathore et al., 2014; Fan et al., 2017; Lee et al., 2017). The reactions of triglyceride with DMC to produce FAME and GLC instead of glycerol are shown in Figure 1. Dimethyl carbonate is a versatile, eco-friendly, non-corrosive, and non-toxic chemical (Dhawan and Yadav, 2017), which is usually produced via oxidative carbonylation of methanol (Zhou et al., 2015). A maximum FAME yield of 96.2% was reported (Zhang et al., 2010) for transesterification at 9:1 DMC to palm oil molar ratio, 8.5 wt% KOH catalyst, 8 h reaction time, and 75°C temperature.

It has also been shown that ≥96% FAME yield was achieved after 45 min for transesterification of jatropha and pongamia oils with DMC or diethyl carbonates under supercritical conditions of 325°C and 150 bar at 40:1 molar ratio of DMC or diethyl carbonate to oil (Rathore et al., 2014). Enzymatic catalysis has been shown to be suitable for triglyceride transesterification with DMC (Seong et al., 2011; Lee et al., 2017). This method was used to achieve optimal yields of 96.3% FAME and 99.7% GLC after 48 h, for reactions at 9.27:1 DMC to soybean oil molar ratio, 52.56°C reaction temperature, and 116.76 g L^−1^ of enzyme concentration in the reaction mixture (Lee et al., 2017). Another study also used enzyme catalysis to achieve maximum conversions of 84.9% biodiesel and 92.0% GLC after 48 h, for transesterification at 6:1 DMC to soybean oil molar ratio, 60°C reaction temperature, and 100 g L^−1^ Novozym 435 enzyme concentration, with tert-butanol as a solvent (Seong et al., 2011). A glycerol-free biodiesel production has been reported elsewhere, where maximum FAME yield of 95.7% was obtained after 5 h for reactions at 5:1 DMC to rapeseed oil (RSO) molar ratio and 110°C temperature, using 25 wt% of sulfonated imidazolium ionic liquid as catalyst (Fan et al., 2017). A triglyceride transesterification using a combination of both methanol and DMC have been reported (Dhawan and Yadav, 2017), where 97.3% conversion of soybean oil and 93.2% selectivity of GLC after 3 h were obtained, for reactions at 90:30:1 of methanol-DMC-soybean oil molar ratio, 150°C temperature, and 12.5 g L^−1^ of hydrotalcite catalyst loading.

The existing biodiesel production methodologies using DMC have inherent severe operational disadvantages arising from the high reaction temperatures (Rathore et al., 2014; Dhawan and Yadav, 2017; Fan et al., 2017), long reaction times (Zhang et al., 2010; Seong et al., 2011; Fan et al., 2017; Lee et al., 2017), and requirements of excessively large molar ratios of DMC and DMC/methanol (Rathore et al., 2014; Dhawan and Yadav, 2017). These technical setbacks must be overcome before triglyceride transesterification with DMC could be considered of process economic advantage compared to the present conventional biodiesel technology. The use of large excesses of DMC and DMC/methanol in the existing studies is uneconomical, and this would particularly be counter-productive to the desire to achieve process advantage by producing biodiesel and valorising the crude glycerol *in situ* to form GLC.

A process simulation and economic analysis of biodiesel production have shown that the amount of energy required for excess methanol recovery was about 23% of the total energy consumption in a conventional biodiesel production reaction using 6:1 methanol to oil molar ratio (Lee et al., 2011). The energy requirement proportionally increases to about 37% and 46% of the total energy consumption of the conventional biodiesel process for 9:1 and 12:1 methanol to oil molar ratios, respectively. Therefore, the existing methods for biodiesel and GLC co-productions are very expensive due to use of energy-intensive operational conditions, and in some cases, partly due to costly enzymatic catalysts. This makes it difficult to commercialize such strategies as they may not compare favorably with the conventional biodiesel process.

The aim of this work was to develop a novel biodiesel process by the application of *in situ* reactive coupling to produce FAME and GLC. It is envisaged that this process would run on less than stoichiometric methanol requirement of 3:1, where small amounts of methanol could be applied to initialize the triglyceride transesterification step. The presence of DMC would convert the reactively-formed crude glycerol into GLC, a highly valuable product, and the reactions of the DMC with glycerol would generate methanol to sustain the triglyceride transesterification. Base-catalyzed triglyceride transesterification is essentially rapid at 60°C, atmospheric pressure, and methanol to oil molar ratio of 3:1, however, the equilibrium FAME conversion is limited to <80% (Eze et al., 2014). Therefore, reactive coupling of the triglyceride transesterification with DMC would accelerate the reactions at moderate process conditions, whereas equilibrium limitation in the FAME conversions would be substantially eliminated by transformations of the reactively-formed glycerol to form GLC. This strategy would potentially allow for use of less methanol and DMC, and operations at moderate reaction conditions. Reactive coupling of the triglyceride transesterification to FAME with *in situ* valorisation of the crude glycerol to GLC at ~60°C, atmospheric pressure, and DMC/methanol to oil molar ratios <6:1 required in the biodiesel conventional process is paramount to economic viability and acceptability in the biodiesel industry. TBD guanidine catalyst was selected for the experimental investigation to avoid soap formations due to the low methanol molar ratio envisaged. The TBD guanidine catalyst does not cause triglyceride and FAME saponification side reactions (Schuchardt et al., 1995).

## Experimental Methods

### Materials

Anhydrous methanol (99.8% purity), DMC (99% purity), triazabicyclodecene guanidine (99% purity), acetic acid (99% purity), 2-propanol (99.5% purity), methyl heptadecanoate (99.0% purity), and GLC (90% purity) were purchased from Sigma-Aldrich, whilst the RSO used was supplied from Henry Colbeck Ltd, UK. The RSO used in the experiments contained ≥99 wt% triglycerides, 0.06 wt% FFA (oleic acid) and 0.01 wt% water.

### Experimental Procedure

The experiments were carried out using a 250 mL sealed batch reactor equipped with a temperature-controlled hot plate magnetic stirrer (IKA® RCT basic IKAMAG™ safety control). The required amount of RSO, methanol and DMC (20 mL total volume) was heated in the batch reactor to the reaction temperature (50–80°C), followed by the addition of 5 wt.% TBD-guanidine based on the RSO. The reaction mixture was mixed vigorously using the magnetic stirrer at 600 rpm to ensure that the reaction was mass transfer independent, based on existing studies on homogenous biodiesel production reactions (Noureddini and Zhu, 1997; Vicente et al., 2005; Eze et al., 2014). The ranges of process parameters studied were methanol-to-RSO molar ratios from 0:1 to 6:1, DMC-to-RSO molar ratios from 1:1 to 6:1, and reaction temperatures of 50–80°C. These range of process parameters were screened using Design of Experiments, response surface methodology. Known amounts (about 1 mL) of samples were collected from the reaction mixture at time intervals from 1 min to 2 h and quenched using calculated amounts of acetic acid. These samples were analyzed using a gas chromatograph (GC) to monitor the extents of conversions of the RSO to FAME and GLC were monitored. The use of 2 h reaction time was based on preliminary results which showed that this period is sufficient to reach the equilibrium conversions. The product samples settled into two distinct phases in the reactions where methanol was used, with upper layers consisting mainly of biodiesel/DMC and a lower layer containing GLC, residual glycerol, methanol, and catalyst. Statistical significance for the investigated parameters were analyzed using the Minitab 17 statistical software at a significance level of 0.05, corresponding to confidence level of 95%. Therefore, the effects of the reaction parameters on the FAME and GLC yields were considered to be statistically significant at *p*-values < 0.05, otherwise, a null hypothesis was returned, and the studied parameters are not statistically significant (*p* ≥ 0.05).

### Analytical Methods

The collected samples were homogenized by adding a known weight (0.5 mL) of 2-propoanol to obtain uniform mixtures for the GC quantifications of the FAME and GLC conversions. About 50–80 mg of the homogenized sample was measured into a 2 mL GC vial, followed by the addition of 1 mL of a 10 mg mL^−1^ of methyl heptadecanoate prepared in in a 2-propanol. The prepared samples were analyzed using a 6890 Hewlett Packard gas chromatograph by injection of 1 μL of sample with a 5 μL SGE GC syringe. The GC was equipped with a fused silica capillary column of 30 m length, 0.32 mm internal diameter, and film thickness of 0.25 μm. The GC oven temperature programme was: 120°C held for 5 min initially and ramped from 120°C to 260°C at a heating rate of 15 °C /min, and held for another 15 min. The injector and flame ionization detector (FID) temperatures were set at 250 °C and 260 °C, respectively. FAME content in the samples were quantified using the BS EN 14103:2003 (BSI, 2003), whereas the GLC was quantified using a calibration data which were obtained from the response factors of the solutions of GLC and the methyl heptadecanoate standard prepared in a 2-propanol. The yields of FAME and GLC were calculated using Equations (1) and (2), respectively. Fatty acid profile of the produced FAME was obtained using Equation (3) and compared with the fatty acid profile of the RSO feedstock, as shown in Table 1. The fatty acid profiles of the FAME and RSO were similar, indicating no modification in the fatty acid composition of the RSO by the reactively-coupled transesterification process.

(1) $$\begin{array}{l} FAME\;yield\;\left(\%\right)=\;\frac{FAME\;content\;of\;the\;sample\;}{Maximum\;theoretical\;FAME}*100 \end{array}$$

(2) $$\begin{array}{l} \text{GLC yield}\left(\%\right)=\;\frac{GLC\;content\;of\;the\;sample\;}{Maximum\;theoretical\;GLC}*100 \end{array}$$

(3) $$\begin{array}{l} Fatty\;acid\;content\;\left(\%\right)=\\ \frac{Peak\;area\;of\;a\;specific\;FAME\;}{Total\;peak\;areas\;of\;all\;FAMEs\;in\;the\;sample}*100 \end{array}$$

**Table 1:** Fatty acid profile of the RSO feedstock and the FAME obtained from reactively coupled biodiesel and GLC productions.

**Type of fatty acids**	**Molecular formula of the fatty acids** **(C_**n**_H_**2n+1**_ COOH)**	**Fatty acids profile (wt. %) for the RSO^a^**	**FAME fatty acids** **profile (wt. %) for biodiesel-GLC**
Palmitic (C16:0)	C_15_H_31_COOH	4.71	5.12
Stearic (C18:0)	C_17_H_35_COOH	1.52	1.62
Oleic (C18:1)	C_17_H_33_COOH	61.47	60.92
Linoleic (C18:2)	C_17_H_31_COOH	19.79	19.14
Linolenic (C18:3)	C_17_H_29_COOH	9.26	9.97
Arachidic (C20:0)	C_19_H_39_COOH	0.59	0.53
Icosenoic (C20:1)	C_19_H_37_COOH	1.37	1.62
Behenic (C22:0)	C_21_H_43_COOH	0.32	0.26
Erucic (C22:1)	C_21_H_41_COOH	0.57	0.80

a *RSO fatty acid profile from Henry Colbeck Ltd. product datasheet*.

## Results and Discussion

### Trend in the Process Parameters

The effects of the process parameters on the formations of FAME and GLC at the reaction conditions of methanol-to-oil molar ratios from 0:1 to 2:1, DMC-to-oil molar ratios from 1:1 to 3:1, and reaction temperatures of 50–80°C are shown in Table 2, indicating average values of FAME and GLC yields and the standard errors from duplicate experiments. The results clearly demonstrate that the equilibrium yields in absence of methanol were 70–82% for FAME and 56–78.8% for GLC for the reactions at 50–80°C, and 1–3: 1 molar ratios of DMC to RSO. This indicates that without methanol, high (≥90) FAME and GLC yields cannot be achieved at moderate reaction temperatures, which is consistent with findings elsewhere (Zhang et al., 2010), where <40% FAME yield was reported at 3:1 DMC to palm oil molar ratios, 75°C temperature, and 8.5% KOH catalyst. The reported lower FAME yield of <40% could be due to triglyceride and FAME saponification side reactions which usually occurs at large alkali metal hydroxide (8.5 wt% KOH) concentrations (Phan et al., 2012; Eze et al., 2014, 2018). The use of TBD guanidine in this study prevented such deleterious side reactions.

**Table 2:** FAME and GLC yields from duplicate experiments at various reaction conditions for the design of experiment.

**Methanol/RSO molar ratio**	**DMC/RSO molar ratio**	**Temperatures (^°^C)**	**Average FAME yields (%)**	**Average GLC yields (%)**
1	2	65	90.0 ± 4.1	70.0 ± 4.0
0	3	65	79.8 ± 3.1	77.6 ± 3.7
1	2	65	90.1 ± 3.8	68.9 ± 1.6
1	3	80	84.0 ± 1.7	79.0 ± 4.2
1	1	50	93.3 ± 1.8	57.6 ± 3.3
2	2	80	97.0 ± 1.6	91.0 ± 4.2
1	3	50	98.0 ± 1.8	76.7 ± 3.8
2	1	65	89.6 ± 4.8	66.8 ± 5.4
2	2	50	98.0 ± 1.5	75.0 ± 5.5
0	2	80	82.0 ± 1.9	78.8 ± 6.8
0	2	50	73.0 ± 3.0	56.0 ± 6.5
2	3	65	98.0 ± 2.6	86.8 ± 5.4
1	2	65	90.0 ± 5.2	72.7 ± 3.8
0	1	65	70.0 ± 1.8	65.5 ± 4.9
1	1	80	84.0 ± 4.2	70.9 ± 6.2
3^a^	3	60	97.6 ± 2.8	91.7 ± 4.6
6^a^	3	60	98.4 ± 2.0	73.3 ± 6.0
2^a^	6	60	98.1 ± 3.1	94.1 ± 2.8

a *Experiments outside the design of experiment space*.

Additions of 1–2:1 methanol to RSO molar ratios substantially enhanced the FAME and GLC yields, as these increased to 84–98% for FAME and 68.9–91% for the GLC for reactions at 2–3:1 DMC-to-RSO molar ratios and 50–80°C temperatures. The data in Table 2 were analyzed using a response surface model in Minitab statistical software to obtain empirical models shown in Equation (4) for the FAME yields and Equation (5) for the GLC, where X_1_ is methanol/ RSO molar ratio, X_2_ is DMC/RSO molar ratio, and X_3_ is reaction temperature (°C).

(4) $$\begin{array}{l} \text{FAME yield}\left(\%\right)=80.3+30.4{\text{X}}_{1}+16.2{\text{X}}_{2}-0.77{\text{X}}_{3}-3.98{\text{X}}_{1}^{2}\\ \;-1.66{\text{X}}_{2}^{2}+0.0077{\text{X}}_{3}^{2}-0.36{\text{X}}_{1}{\text{X}}_{2}-0.183{\text{X}}_{1}{\text{X}}_{3}\\ \;-0.095{\text{X}}_{2}{\text{X}}_{3} \end{array}$$

(5) $$\begin{array}{l} \text{GLC yield}\left(\%\right)=14.7-0.7{\text{X}}_{1}+19.0{\text{X}}_{2}+0.39{\text{X}}_{3}+4.04{\text{X}}_{1}^{2}\\ \;-0.41{\text{X}}_{2}^{2}+0.0040{\text{X}}_{3}^{2}+1.97{\text{X}}_{1}{\text{X}}_{2}-0.096{\text{X}}_{1}{\text{X}}_{3}\\ \;-0.183{\text{X}}_{2}{\text{X}}_{3} \end{array}$$

The experimental and the model data for the FAME and GLC yields indicated a high level of agreement between the experimental and predicted data. The *p*-values for the effects of methanol, DMC and temperature on FAME yield were 0.03, 0.175, and 0.305. This showed that only methanol had a significant effect (*p* < 0.05) on the FAME yield (Lee et al., 2017), whereas DMC and temperature had no significant effects on the FAME yields at the reaction temperature of 50–80°C. The non-significant effect of DMC and temperature in the studied range is because near-equilibrium FAME yields were obtained at low levels of DMC (1:1 DMC to RSO molar ratio) and at 50°C, such that further increases in DMC did not have any significant effect on FAME yield. The *p*-values for the effects of methanol, DMC and temperature on the GLC yield were 0.033, 0.008, and 0.012, respectively (*p* < 0.05 for all parameters), indicating that all the reaction parameters investigated had significant effects on the GLC yields (Lee et al., 2017).

### Process Parameters Interactions and Optimal Fame Yields in a Reactive Coupling

**Figure 2:**
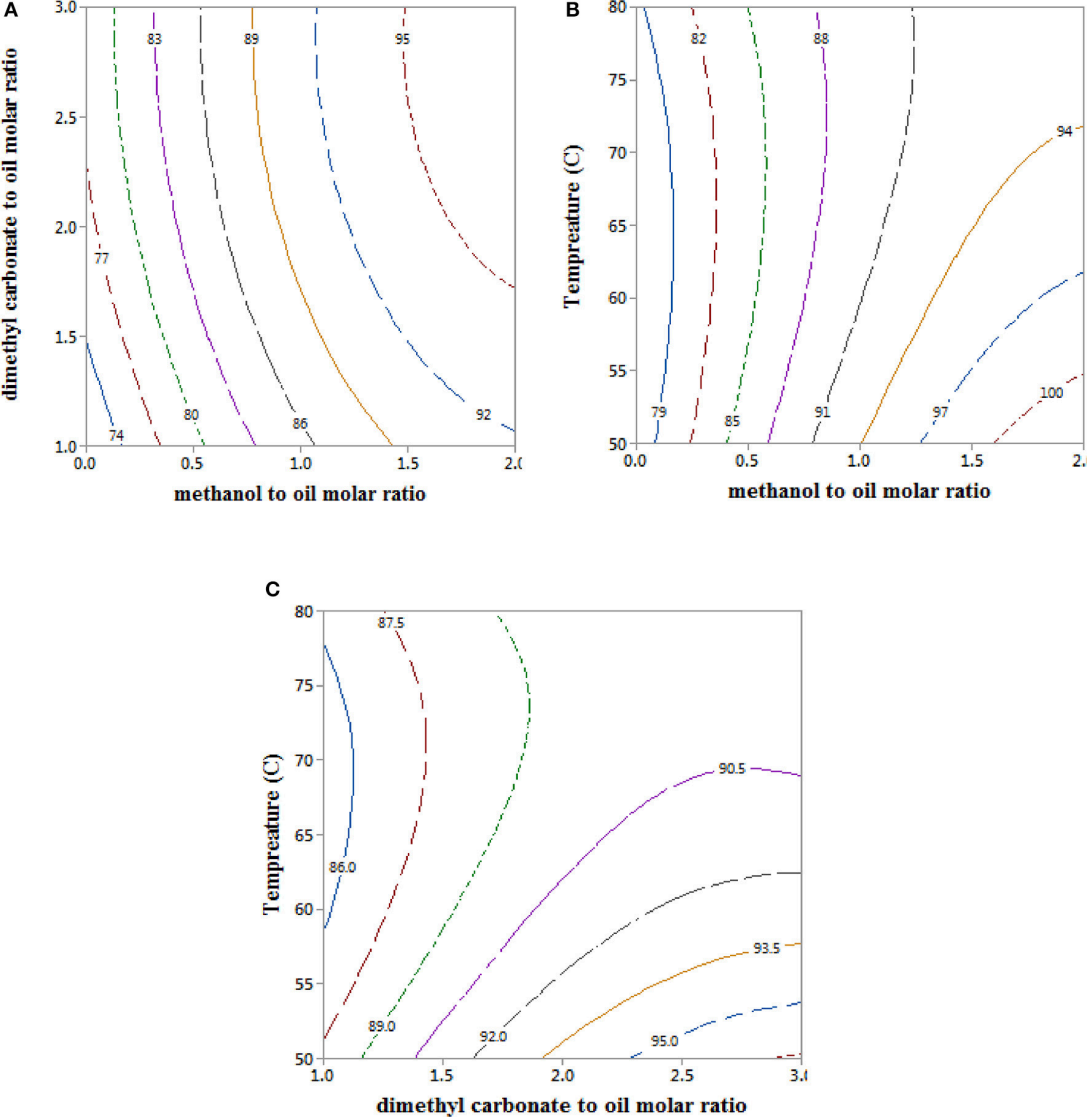
Contour plots for the effect of DMC, methanol, and temperature on the FAME yields for reactive coupling of RSO transesterification with *in situ* crude glycerol valorisation to GLC. **(A)** Interaction between DMC and MeOH to RSO molar ratio at 65°C, **(B)** interaction between temperature and methanol to RSO molar ratio at 2DMC and **(C)** interaction between temperature and DMC at 1 MeOH.

Interaction plots for the process parameters in the empirical model for the FAME yield are shown in Figure 2. It can be seen in the interaction plot between DMC and methanol (Figure 2A) that increase in the methanol to RSO molar ratio resulted in higher FAME yields. For instance, ≥95% FAME yields were obtained after 2 h reaction time at only 2:1 methanol to RSO molar ratio and 2–3:1 DMC to RSO molar ratio, and 65°C temperature. These FAME yields which were achieved at substantially lower DMC to oil and less than stoichiometric requirement of methanol, compares well with the values of 92–97% reported using extreme reaction conditions, such as high reaction temperatures between 110 and 325°C (Rathore et al., 2014; Dhawan and Yadav, 2017; Fan et al., 2017), long reaction times in the range of 5–48 h (Zhang et al., 2010; Seong et al., 2011; Fan et al., 2017; Lee et al., 2017), large molar ratios (40:1) of DMC to oil (Rathore et al., 2014), and excessively large methanol/DMC/oil (90:30:1) molar ratios (Dhawan and Yadav, 2017). Higher DMC to RSO molar ratio also enhanced the FAME yields, as this increased from 75% at 1:1 DMC to RSO molar ratio to 81% at 3:1 DMC to RSO molar ratio, for reactions at 65°C in the absence of methanol. However, the FAME yields increased from 81 to 90% on addition of only 1:1 methanol to RSO molar ratio at the 3:1 DMC to RSO molar ratio and 65°C (Figure 2A).

Clearly, FAME yields ≥95% were obtained at various reaction conditions in the presence of methanol at moderate reaction conditions. It has been reported that 96.2% of FAME yield was obtained after 8 h for transesterification at 6:1 DMC to palm oil molar ratio at refluxing temperatures of 75°C, and using 8.5 wt% KOH catalyst (Zhang et al., 2010), whereas 95.8% FAME yield was achieved after 5 h for transesterification at 5:1 DMC to RSO molar ratio, 110 °C, and 2 wt% of sulfonated imidazolium ionic liquid as a catalyst (Fan et al., 2017). Similar, FAME yields were obtained in this study even at shorter reaction time (1–2 h), DMC to RSO molar ratios of 1–3:1, 1–2:1 methanol to RSO molar ratios and temperature ≤ 80°C. Our findings strongly demonstrate that an optimized reactively coupled triglyceride transesterification and glycerol transformation to GLC is a more efficient strategy for glycerol-free biodiesel process. The reactive coupling strategy is an efficient synergistic process which uses methanol generated from the reaction of DMC with the reactively-formed glycerol to sustain the triglyceride transesterification. As shown in Figures 2B,C, temperature has a very weak effect on the FAME yield within the parameter space investigated. Therefore, it is preferable to operate below 65°C, the ambient pressure boiling point of methanol, as this removes the need for operating at pressure.

### Interactions of Process Parameters and Optimal GLC Yields in a Reactive Coupling

**Figure 3:**
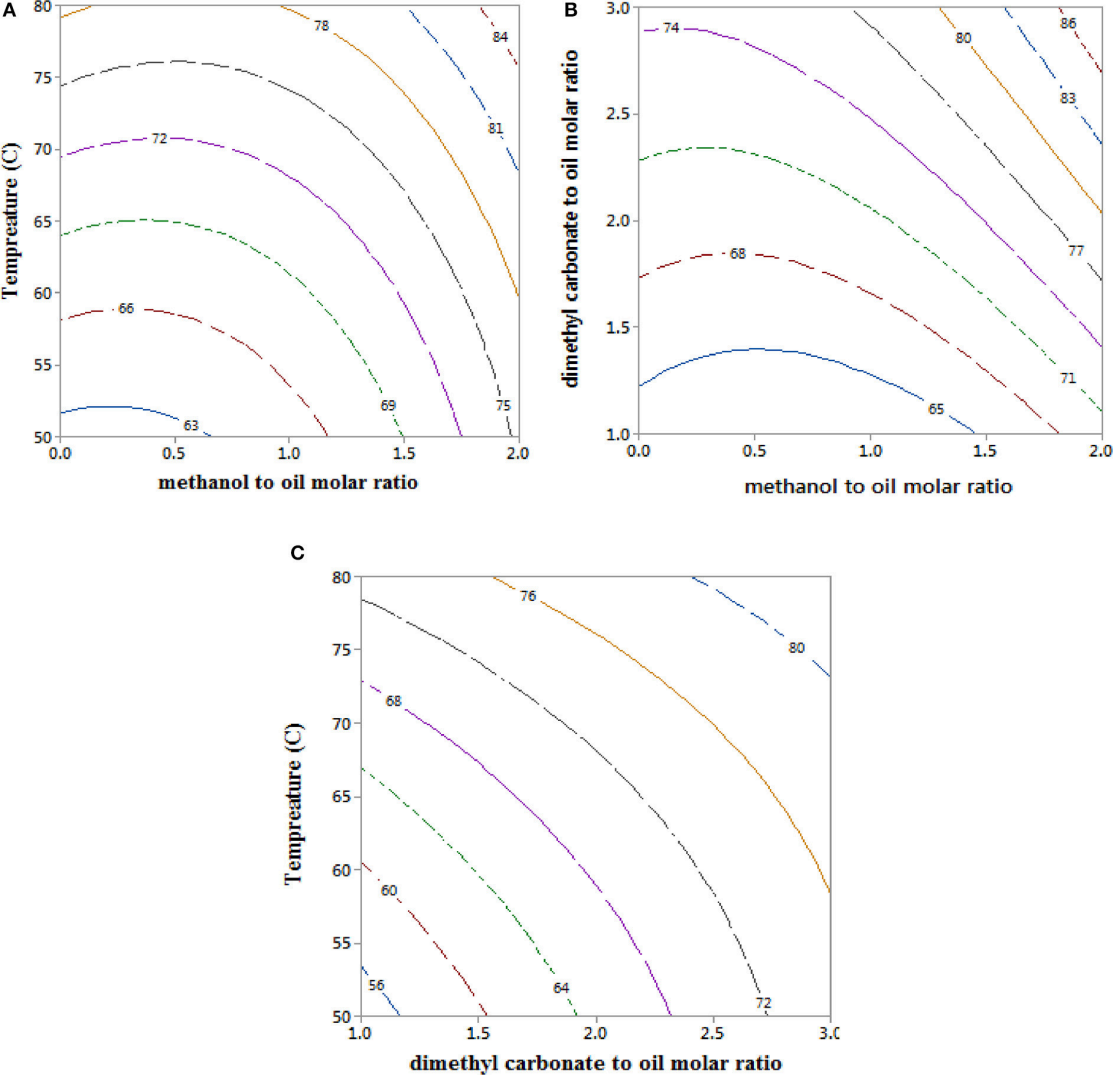
Contour plots for the effect of DMC, methanol, and temperature on the GLC yields for reactive coupling of RSO transesterification with *in situ* crude glycerol valorisation to GLC. **(A)** Interaction between temperature and MeOH at 2 DMC, **(B)** interaction between DMC and MeOH to RSO molar ratio at 65°C and **(C)** interaction between temperature and DMC at 1 MeOH.

Figure 3 shows the interaction effect of operating variables on the GLC yields, indicating that increasing DMC molar ratios (Figure 3A) and reaction temperature (Figure 3C) had positive effects on the GLC yields of GLC. This observation suggests that the GLC formation is more endothermic than the formations of FAME. The observed trends in the GLC yields with the increase DMC and temperature are consistent with existing studies on the effects, of DMC (Zhang et al., 2010; Okoye et al., 2016; Fan et al., 2017) and reaction temperature (Lanjekar and Rathod, 2013; Ishak et al., 2016; Okoye et al., 2016), on GLC formations. It has also been reported that the equilibrium rate constants for GLC formations in the reactions of glycerol with DMC increased from 2.903 at 40°C to 3.81 at 50°C, 4.92 at 60°C, and 6.20 at 70°C (Li and Wang, 2011), indicating a strong interaction between equilibrium GLC yields with the reaction temperatures.

It can be seen from the contour plot in Figure 3B that increasing the methanol to RSO molar ratio led to increased GLC yields, as the presence of methanol in the reaction mixture increased the initial rate of formation of FAME and glycerol by transesterification. This led to higher glycerol concentration in the reaction mixture, and consequently increased rates of reaction with DMC to form GLC. However, at methanol to RSO molar ratios above 3:1, there was a substantial decline in the GLC yield (Table 2), which was attributed to the effect of reverse reaction between GLC and methanol. For instance, the GLC yields decreased from about 90.1–91.7% at 2:1–3:1 methanol-to-RSO molar ratio to 73.3% at 6:1 methanol molar ratio, for reactive coupling at 3:1 DMC-to-RSO molar ratio and 60°C temperature using the TBD guanidine catalyst. Although methanol is required to accelerate the RSO transesterification part of the reaction, excess of methanol in the reaction system leads to lower equilibrium GLC conversion due to thermodynamic equilibrium limitations.

**Figure 4:**
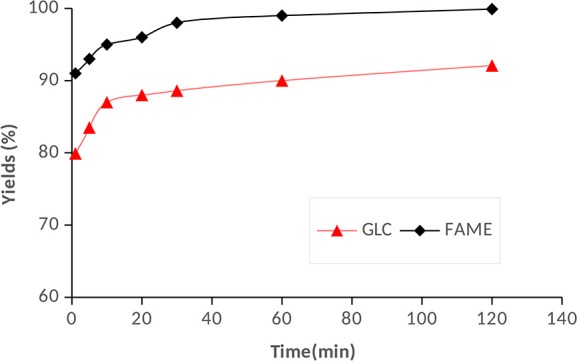
FAME and GLC yields for reactive coupling of RSO transesterification with 2:1 methanol to RSO molar ratio, 3:1 DMC to RSO molar ratio, 60°C reaction temperature, and 5 wt% TBD guanidine.

Additionally, it was observed that additions of methanol resulted in separation of reaction product into two distinct phases of FAME-rich and GLC-rich layers which allows for ease of separation. In absence of methanol, the GLC formed dissolved in the DMC and FAME to form one phase, which would incur greater downstream separation costs. The optimized reaction conditions by response surface method analysis of the experimental data, showed that high FAME and GLC yields could be achieved at methanol to RSO molar ratio of 2:1 and 3:1 DMC to RSO molar ratio at 60°C. The predicted optimal reaction condition was validated as shown in Figure 4, and the results showed that about 90% FAME and 79.9% GLC conversion can be obtained after only 1 min reaction time. The products yield increased to 98.0 ± 1.5% for FAME and 90.1 ± 2.2% for GLC after 60 min, whereas the equilibrium conversions after 2 h reaction time were about 99 and 92% for FAME and GLC, respectively. At the optimized equilibrium FAME and GLC conversions, the upper layer after separation contained mainly of FAME (~85 wt%) and unreacted DMC (~14 wt%). There was a negligible amount of GLC (≤0.5 wt.%) and no glycerol was detected in the upper layer after separation. The bottom layer contained mainly of GLC (60.8 wt%), glycerol (4.3 wt%), FAME (9.6 wt %), and about one mole equivalent of unreacted methanol (16.5 wt%). Therefore, the GLC produced can be relatively easily recovered from the bottom layer through distillation and the unreacted methanol recycled into the process. The energy cost for unreacted methanol recovery in this study is substantially lower than required for 3 moles of unreacted methanol that remains for the conventional base-catalyzed biodiesel production using 6:1 methanol-to-oil molar ratio (Lee et al., 2011). Therefore, apart from the productions of highly valuable GLC, the reactive coupling process requires less methanol, and reduces the cost of unreacted methanol recovery by over 60%. The unreacted DMC in the FAME at the optimal condition was 14 wt%, which is within the range of 1–20 vol% DMC that has been reported to be suitable in reducing smoke opacity and NOx emissions substantially when blended in diesel fuels (Zhang et al., 2005; Rounce et al., 2010). Therefore, it is envisaged that the unreacted DMC would not be separated, as the combustion characteristics of the biodiesel would be improved by the presence of DMC, an oxygenate fuel additive (Zhang et al., 2005; Rounce et al., 2010; Fan et al., 2017).

### Proposed Reaction Scheme in the Reactive Coupling for FAME and GLC Formations

**Figure 5:**
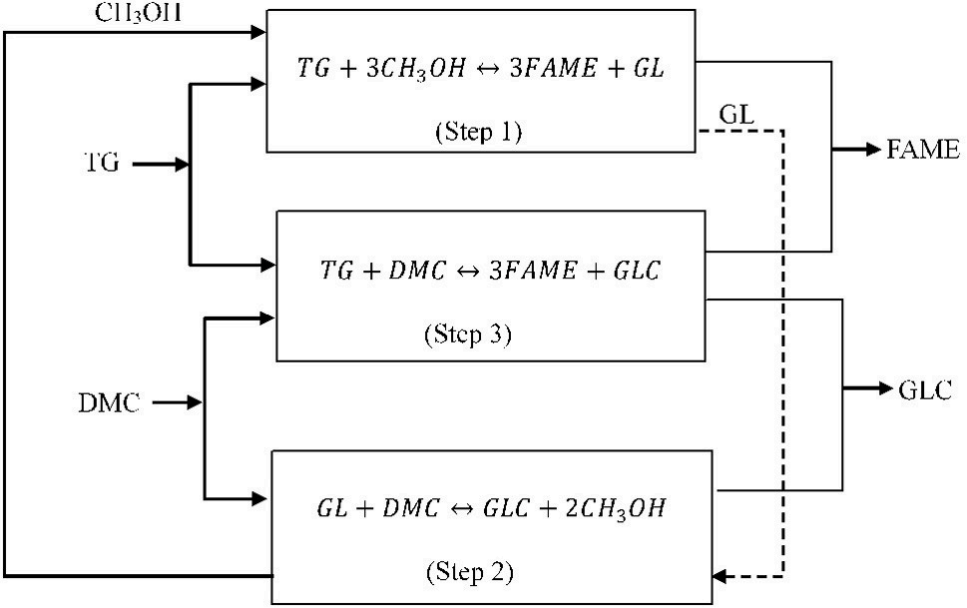
Proposed reaction scheme for triglyceride transesterification with methanol and *in situ* reactive coupling of the by-product glycerol with DMC to form GLC.

Based on the findings from this study and understandings of the biodiesel reaction process, a reaction scheme in Figure 5 has been proposed for the base-catalyzed triglyceride transesterification with *in situ* transformation of the reactively-formed glycerol to GLC. The reactive coupling is a particularly complex chemical process as illustrated by the reaction scheme. The reaction is initiated by generation of methoxide ion (CH_3_O^−^) by the interaction of methanol and base catalyst such as the TBD guanidine, as shown in Equation (6). The methanol in the system reacts with the triglyceride (TG) to form FAME and glycerol (GL) as shown in the Figure 5 (Step 1), through the interactions of the CH_3_O^−^ catalytic species with the TG.

(6) $$\begin{array}{l} TBD+CH_{3}OH\;↔CH_{3}O^{-}+TBDH^{+} \end{array}$$

The glycerol by-product is continually removed from the transesterification reaction equilibrium, by reactive coupling with DMC to from GLC (Figure 5, Step 2), which generates two moles methanol for every one mole of glycerol converted to GLC. The generated methanol is deprotonated by the base catalyst to form more CH_3_O^−^ which sustains the triglyceride transesterification side of the reactive coupling. The diglyceride (DG) and monoglyceride (MG) intermediates can also be removed from the transesterification equilibrium reaction by reacting with DMC.

**Figure 6:**
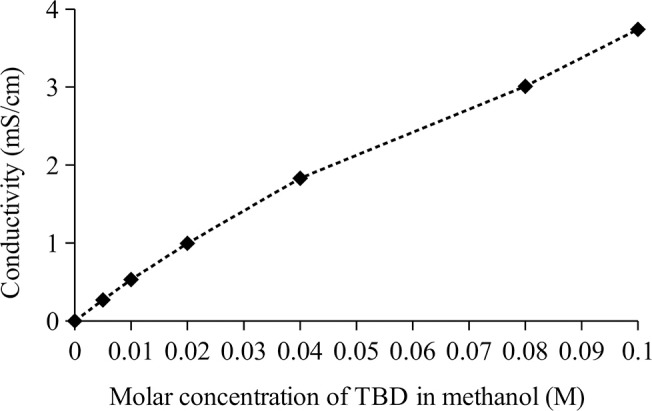
Conductivity of methanol at different TBD guanidine molar concentrations.

The proposed reaction scheme involves formations of CH_3_O^−^ catalytic species via deprotonation of methanol by the TBD guanidine catalyst, as shown in Equation (6). In view of the superbasicity of the TBD, with a pKa of about 25.5 in aprotic solvents (Pratt et al., 2006), deprotonation of methanol by TBD was expected. It has been reported elsewhere that TBD functions by deprotonating or activating alcohols for nucleophilic attack on the substrates during reactions (Kaljurand et al., 2005). However, no experimental data exists to show whether the mode of catalysis was via alkoxide nucleophilic attack from deprotonated alcohol. Measurements of the conductivities of TBD/methanol and TBD/DMC of different TBD molar concentrations were used in this work to investigate any change in ionic conductivity, which would be expected if there are formations of methoxide ions. The conductivities of the TBD solutions were measured using CDM210 conductivity meter (Radiometer Analytical) at 16.8°C. There was a huge rise in conductivity of methanol in the presence of TBD as shown in Figure 6. When TBD was dissolved in DMC, there was a negligible change in conductivities, with 0.00 mS/cm for DMC only (0.0M TBD) and 0.16 mS/cm for 0.1M TBD in DMC.

The high levels of conductivity of TBD/methanol solutions are consistent with formations of methoxide ions. As shown in Figure 6, the conductivities were 0.00 mS/cm for methanol only (0.0M TBD), rising to 3.74 mS/cm for 0.1M TBD in methanol. The change in the methanol conductivity with TBD concentration follows a general trend in conductivity for formations of ions from weak acids and bases due to equilibrium limitations (Martínez, 2018).

Generally, the triglyceride transesterification process occurs via three consecutive step-wise reversible reactions (Mittelbach and Trathnigg, 1990; Darnoko and Cheryan, 2000; Vicente et al., 2005), which are shown Equations (7)–(9). However, in the reactively-coupled transesterification process for simultaneous productions of FAME and GLC, these conventional equilibrium reactions are disrupted. The removal of DG, MG, and GL through reactive coupling with DMC greatly minimizes the reverse reactions in the TG transesterification. The reactive coupling process has enormous advantage, such that use of methanol above the stoichiometric molar ratio of 3:1 would no longer be required. This ensures very efficient methanol utilization and hugely reduces the cost of methanol recovery in the downstream process in biodiesel plants.

(7) $$\begin{array}{l} TG+CH_{3}OH\;↔FAME+DG \end{array}$$

(8) $$\begin{array}{l} DG+CH_{3}OH\;↔FAME+MG \end{array}$$

(9) $$\begin{array}{l} MG+CH_{3}OH\;↔FAME+GL \end{array}$$

As shown in the Figure 5 (Step 3), triglyceride transesterification also occurs with only DMC. However, this reaction was found to be more energy intensive, and requires high reaction temperature to achieve high FAME and GLC conversions. Therefore, reliance on the reactions in Step 3 for co-productions of FAME and GLC would not be economical due to its energy intensive operation. This study strongly demonstrated that high FAME and GLC conversions are only possible through optimized reactive coupling of the triglyceride transesterification and *in situ* glycerol transformation to GLC. To achieve high FAME and GLC yields, the use of methanol must be optimized. It was experimentally observed that use of > 3:1 methanol to RSO molar ratios resulted in lower GLC yields, although the FAME yields were not affected. This observation was attributed to the reverse reaction of glycerol and DMC shown in Figure 5 (Step 2), in which excess methanol could lead to shift in the equilibrium conversions toward glycerol according to the Le Chatelier's principle. Optimal values of 3:1 DMC to oil molar ratio is recommended for the reactive coupling. Although use of DMC above 3:1 DMC to RSO molar ratio did not have any adverse effects on the equilibrium FAME and GLC conversions, the reaction rates were proportionately slowed down due to dilution of the reacting mixtures by excess DMC. Therefore, this study hypothesizes that less than molar stoichiometric amount of methanol (2:1) is required to initialize the triglyceride transesterification side of the reactive coupling, while 3:1 of DMC to oil will be required for the *in situ* glycerol conversions to GLC, to achieve both high FAME and GLC yields at much more favorable and moderate process conditions.

## Conclusions

This study investigated a reactive coupling reaction for optimal transesterification of RSO to FAME and GLC in a one-step process, and at operating conditions which are compatible with current biodiesel industry. The reactive coupling process was studied at various molar ratios of both methanol and DMC to the triglyceride [rapeseed oil (“RSO”)], using a TBD guanidine catalyst and reaction temperatures of 50–80°C. The optimal reaction conditions identified, using a Design of Experiments approach, were a 2:1 methanol-to-RSO molar ratio and 3:1 DMC -to-RSO molar ratio at 60°C. The FAME and GLC conversions at these conditions were 98.0 ± 1.5% and 90.1 ± 2.2%, respectively, after 1 h reaction time using the TBD guanidine catalyst. Increasing the DMC-to-RSO molar ratio from 3:1 to 6:1 slightly improved the GLC conversion to 94.1 ± 2.8% after 2 h, but did not increase FAME conversion. Methanol substantially improved both FAME and GLC conversions at 1:1–2:1 methanol-to-RSO molar ratios and enhanced the GLC separation from the reaction mixture. It was observed that higher methanol molar ratios (>3:1) enhanced only FAME conversions, with the excess methanol resulting in lower GLC conversions, achieving 73.3% GLC yield for a 6:1 methanol-to-RSO molar ratio, a DMC-to-RSO molar ratio of 3:1 and 60°C. This study clearly demonstrates that formation of the low value crude glycerol in conventional biodiesel processing can be greatly reduced, by over 90%, with proportionate formation of GLC as a more valuable product. This biodiesel production through reactive coupling minimizes the methanol requirement, whilst simultaneously producing GLC, thereby improving the economics of biodiesel production, and rendering the process “greener,” as crude glycerol waste is reduced.

## Author Contributions

AH and VE designed the experimental programme, and the experimental data were collected by LA-S. Initial draft of the manuscript was written by LA-S, VE. The overall supervisions of the experimental work and review of the manuscript were carried out by AH.

### Conflict of Interest Statement

The authors declare that the research was conducted in the absence of any commercial or financial relationships that could be construed as a potential conflict of interest.

## References

[B1] Ashby R. Solaiman D. Y. Foglia T. (2004). Bacterial poly(Hydroxyalkanoate) polymer production from the biodiesel co-product stream. J. Polymers Environ. 12, 105–112. 10.1023/B:JOOE.0000038541.54263.d9

[B2] Ayoub M. Abdullah A. Z. (2012). Critical review on the current scenario and significance of crude glycerol resulting from biodiesel industry towards more sustainable renewable energy industry. Renew. Sustain. Energy Rev. 16, 2671–2686. 10.1016/j.rser.2012.01.054

[B3] Bromberg L. Fasoli E. Alvarez M. Hatton T. A. Barletta G. L. (2010). Biguanide-, imine-, and guanidine-based networks as catalysts for transesterification of vegetable oil. Reactive Funct. Polym. 70, 433–441. 10.1016/j.reactfunctpolym.2010.04.003 20495678 PMC2873605

[B4] BSI (2003). Fat and Oil Derivatives - Fatty Acid Methyl Esters (Fame) For Diesel Engines - Determination of Esters and Linolenic Acid Methyl Ester Contents, in Designation BS EN 14103. London.

[B5] Canakci M. Van Gerpen J. (2003). A pilot plant to produce biodiesel from high free fatty acid feedstocks. Trans. Am. Soc. Agri. Eng. 46, 945–954. 10.13031/2013.13949

[B6] Cervero J. M. Coca J. Luque S. (2008). Production of biodiesel from vegetable oils. Grasas y Aceites 59, 76–83. 10.3989/gya.2008.v59.i1.494

[B7] Ciriminna R. Della Pina C. Rossi M. Pagliaro M. (2014). Understanding the glycerol market. Eur. J. Lipid Sci. Technol. 116, 1432–1439. 10.1002/ejlt.201400229

[B8] Darnoko D Cheryan M. (2000). Kinetics of palm oil transesterification in a batch reactor. J. Am. Oil Chem. Soc. 77, 1263–1267. 10.1007/s11746-000-0198-y

[B9] Demirbas M. F. Balat M. (2006). Recent advances on the production and utilization trends of bio-fuels: a global perspective. Energy Conv. Manag. 47, 2371–2381. 10.1016/j.enconman.2005.11.014

[B10] Derrien A. Renard G. Brunel D. (1998). Guanidine linked to micelle-templated mesoporous silicates as base catalyst for transesterification. Studies Surf. Sci. Catal. 117, 445–452. 10.1016/S0167-2991(98)81023-9

[B11] Dhawan M. S. Yadav G. D. (2017). Insight into a catalytic process for simultaneous production of biodiesel and glycerol carbonate from triglycerides. Catalysis Today 309, 161–171. 10.1016/j.cattod.2017.08.020

[B12] Esteban J. Domínguez E. Ladero M. Ochoa F. G. (2015). Kinetics of the production of glycerol carbonate by transesterification of glycerol with dimethyl and ethylene carbonate using potassium methoxide, a highly active catalyst. Fuel Process Technol. 138(Suppl. C), 243–251. 10.1016/j.fuproc.2015.06.012

[B13] Eze V. C. Harvey A. P. (2018). Continuous reactive coupling of glycerol and acetone A strategy for triglyceride transesterification and in-situ valorisation of glycerol by-product. Chem. Eng. J. 347, 41–51. 10.1016/j.cej.2018.04.078

[B14] Eze V. C. Phan A. N. Harvey A. P. (2018). Intensified one-step biodiesel production from high water and free fatty acid waste cooking oils. Fuel 220, 567–574. 10.1016/j.fuel.2018.02.050

[B15] Eze V. C. Phan A. N. Harvey A. P. (2014). A more robust model of the biodiesel reaction, allowing identification of process conditions for significantly enhanced rate and water tolerance. Bioresour. Technol. 156, 222–231. 10.1016/j.biortech.2014.01.028 24508659

[B16] Fan P. Wang J. Xing S. Yang L. Yang G. Fu J. (2017). Synthesis of glycerol-free biodiesel with dimethyl carbonate over sulfonated imidazolium ionic liquid. Energy Fuels. 31, 4090–4095. 10.1021/acs.energyfuels.7b00115

[B17] Huber G. W. Iborra S. Corma A. (2006). Synthesis of transportation fuels from biomass: Chemistry, catalysts, and engineering. Chem. Rev. 106, 4044–4098. 10.1021/cr068360d 16967928

[B18] Ishak Z. I. AsrinaSairi N. Alias Y. Taieb Aroua M. K. Yusoff R. (2016). Production of glycerol carbonate from glycerol with aid of ionic liquid as catalyst. Chem. Eng. J. 297(Suppl. C), 128–138. 10.1016/j.cej.2016.03.104

[B19] Kaljurand I. Kütt A. Sooväli L. Rodima T. Mäemets V. Leito I.. (2005). Extension of the self-consistent spectrophotometric basicity scale in acetonitrile to a full span of 28 pKa units: unification of different basicity scales. J. Org. Chem. 70, 1019–1028. 10.1021/jo048252w 15675863

[B20] Lanjekar K. Rathod V. K. (2013). Utilization of glycerol for the production of glycerol carbonate through greener route. J. Environ. Chem. Eng. 1, 1231–1236. 10.1016/j.jece.2013.09.015

[B21] Lee S. Posarac D. Ellis N. (2011). Process simulation and economic analysis of biodiesel production processes using fresh and waste vegetable oil and supercritical methanol. Chem. Eng. Res. Design. 89, 26262642 10.1016/j.cherd.2011.05.011

[B22] Lee Y. Lee J. H Yanga H. J. Jang M Kim J. R. Byun E. H. (2017). Efficient simultaneous production of biodiesel and glycerol carbonate via statistical optimization. J. Ind. Eng. Chem. 51, 49–53. 10.1016/j.jiec.2017.03.010

[B23] Li J. Wang T. (2011). Chemical equilibrium of glycerol carbonate synthesis from glycerol. J. Chem. Thermodynam. 43, 731–736. 10.1016/j.jct.2010.12.013

[B24] Martínez L. (2018). Measuring the conductivity of very dilute electrolyte solutions, drop by drop. Quimica Nova. 41, 814–817. 10.21577/0100-4042.20170216

[B25] Meloni D. Monaci R. Zedde Z. Cutrufello M. G. Fiorilli S. Ferino I. (2011). Transesterification of soybean oil on guanidine base-functionalized SBA-15 catalysts. Appl. Catal. B Environ. 102, 505–514. 10.1016/j.apcatb.2010.12.032

[B26] Mittelbach M. Trathnigg B. (1990). Kinetics of alkaline catalyzed methanolysis of sunflower oil. Lipid. Fett. 92, 145–148.

[B27] Moser B. (2009). Biodiesel production, properties, and feedstocks. In Vitro Cell. Dev. Biol. Plant 45, 229–266. 10.1007/s11627-009-9204-z

[B28] Mota C. J. A. da Silva C. X. A. Rosenbach N. Costa J. da Silva F. (2010). Glycerin derivatives as fuel additives: the addition of glycerol/acetone ketal (Solketal) in gasolines. Energy Fuels 24, 2733–2736. 10.1021/ef9015735

[B29] Mu Y. Teng H. Zhang D. J. Wang W. Xiu Z. L. (2006). Microbial production of 1,3-propanediol by Klebsiella pneumoniae using crude glycerol from biodiesel preparations. Biotechnol. Lett. 28, 1755–1759. 10.1007/s10529-006-9154-z 16900328

[B30] Nguyen P. T. Nohair B. Mighri N. Kaliaguine S. (2013). TBD-functionalized mesoporous silica: synthesis and catalytic activity in corn oil transesterification. Microporous Mesoporous Materials 180, 293–300. 10.1016/j.micromeso.2013.07.001

[B31] Noureddini H. Zhu D. (1997). Kinetics of transesterification of soybean oil. J. Am. Oil Chem. Soc. 74, 1457–1463. 10.1007/s11746-997-0254-2

[B32] Okoye P. U. Abdullah A. Z. Hameed B. H. (2016). Glycerol carbonate synthesis from glycerol and dimethyl carbonate using trisodium phosphate. J. Taiwan Instit. Chem. Eng. 68(Suppl. C), 51–58. 10.1016/j.jtice.2016.09.011

[B33] Papanikolaou S. Muniglia L. Chevalot I. Aggelis G. Marc I. (2002). Yarrowia lipolytica as a potential producer of citric acid from raw glycerol. J. Appl. Microbiol. 92, 737–744. 10.1046/j.1365-2672.2002.01577.x 11966915

[B34] Phan A. N. Harvey A. P Eze V. (2012). Rapid production of biodiesel in mesoscale oscillatory baffled reactors. Chem. Eng. Technol. 35, 1214–1220. 10.1002/ceat.201200031

[B35] Pratt R. C. Lohmeijer B. G. Long D. A. Waymouth R. M. Hedrick J. L. (2006). Triazabicyclodecene: a simple bifunctional organocatalyst for acyl transfer and ring-opening polymerization of cyclic esters. J. Am. Chem. Soc. 128, 4556–4557. 10.1021/ja060662 16594676

[B36] Quispe C. A. G. Coronado C. J. R. Carvalho J. A. Jr. (2013). Glycerol: Production, consumption, prices, characterization and new trends in combustion. Renewable Sustain. Energy Rev. 27, 475–493. 10.1016/j.rser.2013.06.017

[B37] Rathore V. Tyagi S. Newalkar B. Badoni R. P. (2014). Glycerin-free synthesis of jatropha and pongamia biodiesel in supercritical dimethyl and diethyl carbonate. Ind. Eng. Chem. Res. 53, 10525–10533. 10.1021/ie5011614

[B38] Rodrigues R. Isoda N. Gonçalves M. Figueiredo F. C. A. Mandelli D. Carvalh W. A. (2012). Effect of niobia and alumina as support for Pt catalysts in the hydrogenolysis of glycerol. Chem. Eng. J. 198–199, 457–467. 10.1016/j.cej.2012.06.002

[B39] Rounce P. Tsolakis A. Leung P. York A. P. E. (2010). A comparison of diesel and biodiesel emissions using dimethyl carbonate as an oxygenated additive. Energy Fuels 24, 4812–4819. 10.1021/ef100103z

[B40] Schuchardt U Vargas R. M. Gelbard G. (1995). Alkylguanidines as catalysts for the transesterification of rapeseed oil. J. Mol. Catal. A. Chem. 99, 65–70. 10.1016/1381-1169(95)00039-9

[B41] Seong P. J. Jeon B. W. Lee M. Cho D. H. Kim D. K. Jung K. S.. (2011). Enzymatic coproduction of biodiesel and glycerol carbonate from soybean oil and dimethyl carbonate. Enzyme Microbial Technol. 48, 505–509. 10.1016/j.enzmictec.2011.02.009 22113023

[B42] Sercheli R. Vargas R. M Schuchardt U. (1999). Alkylguanidine-catalyzed heterogeneous transesterification of soybean oil. J. Am. Oil Chem. Soc. 76, 1207–1210. 10.1007/s11746-999-0095-2

[B43] Teng W. K. Ngoh G. C. Yusoff R. Aroua M. K. (2016). Microwave-assisted transesterification of industrial grade crude glycerol for the production of glycerol carbonate. Chem. Eng. J. 284(Suppl. C), 469–477. 10.1016/j.cej.2015.08.108

[B44] Vicente G. Martínez M. Aracil J. Esteban A. (2005). Kinetics of sunflower oil methanolysis. Ind. Eng. Chem. Res. 44, 5447–5454. 10.1021/ie040208j

[B45] Zabeti M. Wan Daud W. M. A. Aroua M. K. (2009). Activity of solid catalysts for biodiesel production: a review. Fuel Process. Technol. 90, 770–777. 10.1016/j.fuproc.2009.03.010

[B46] Zhang G. D. Liu H. Xia X. X Zhang W. G. Fang J. H. (2005). Effects of dimethyl carbonate fuel additive on diesel engine performances. Proc. Insti. Mechan. Eng. Part D J. Automob. Eng. 219, 897–903. 10.1243/095440705X28358

[B47] Zhang L. Sheng B. Xin Z. Liu Q. Sun S. (2010). Kinetics of transesterification of palm oil and dimethyl carbonate for biodiesel production at the catalysis of heterogeneous base catalyst. Bioresour. Technol. 101, 8144–8150. 10.1016/j.biortech.2010.05.069 20591662

[B48] Zhou Y. Wang S. Xiao M. Han D. Lu Y. Menga Y. (2015). Formation of dimethyl carbonate on nature clay supported bimetallic copper-nickel catalysts. J. Cleaner Production 103 (Suppl. C), 925–933. 10.1016/j.jclepro.2014.08.075

